# Supplementary material for: A novel similarity score based on gene ranks to reveal genetic relationships among diseases
Source: PeerJ. 2021 Jan 6;9:e10576. doi: 10.7717/peerj.10576 (PMC7796663; doi:10.7717/peerj.10576)
Supplement: Supplemental Information 1 [file peerj-09-10576-s001.docx]

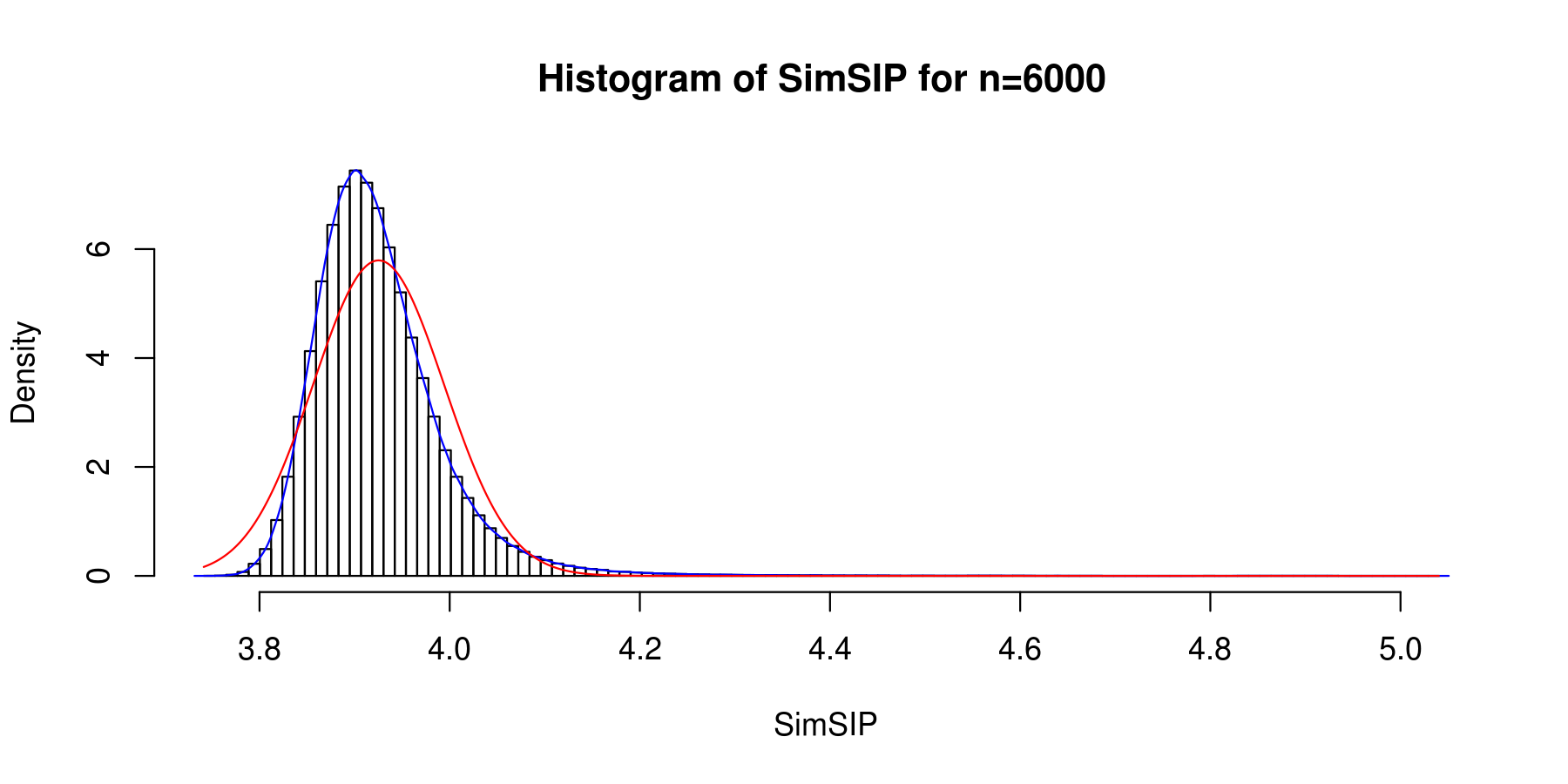


Figure S1 The empirical distribution of $SimSIP$under null hypothesis when S=10millions. The blue curve is the fitting kernel density curve and the red curve is the fitting normal distribution curve.


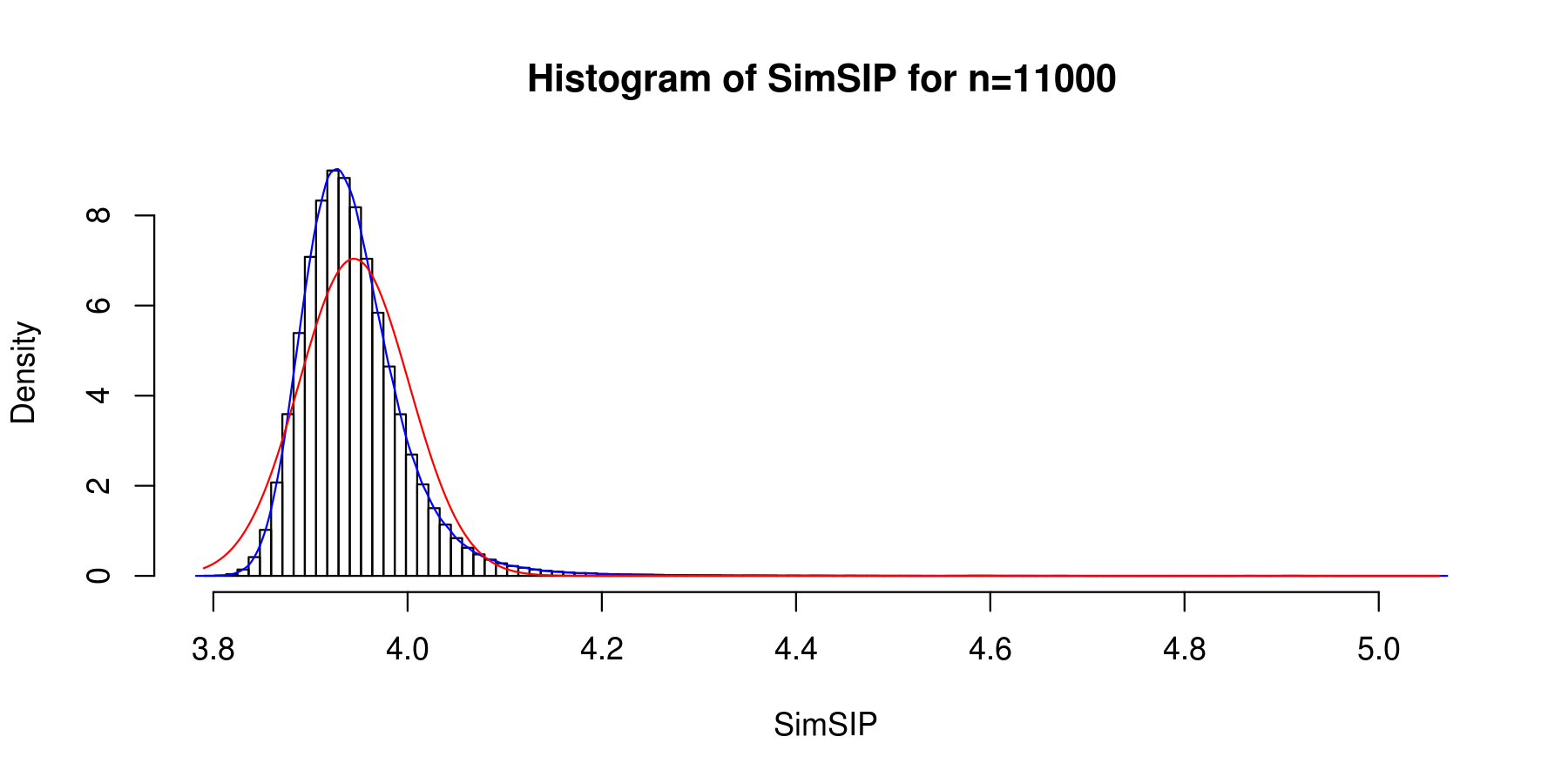


Figure S2 The empirical distribution of$SimSIP$ under null hypothesis when S=10millions. The blue curve is the fitting kernel density curve and the red curve is the fitting normal distribution curve.


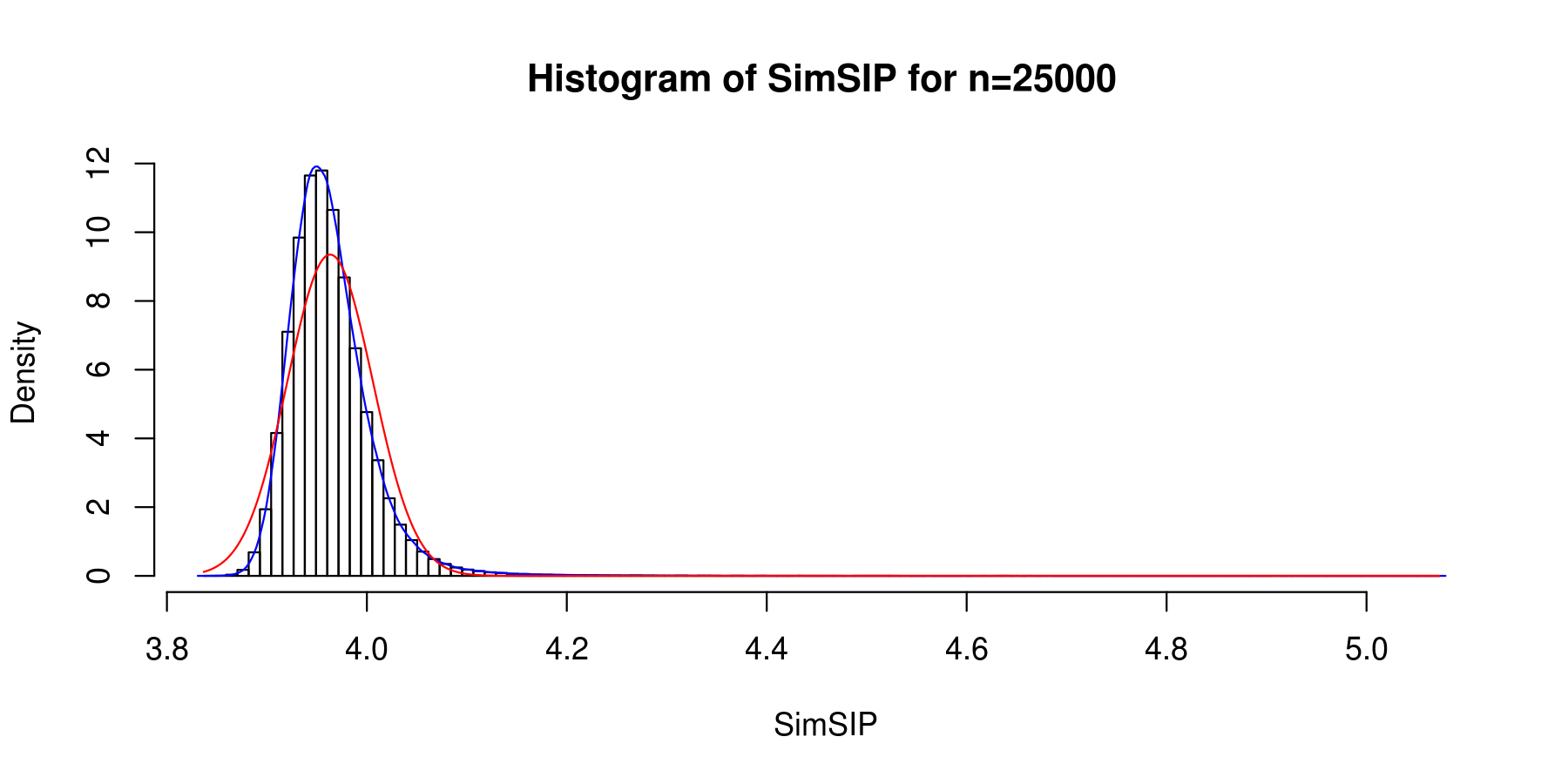


Figure S3 The empirical distribution of $SimSIP$under null hypothesis when S=10millions. The blue curve is the fitting kernel density curve and the red curve is the fitting normal distribution curve.


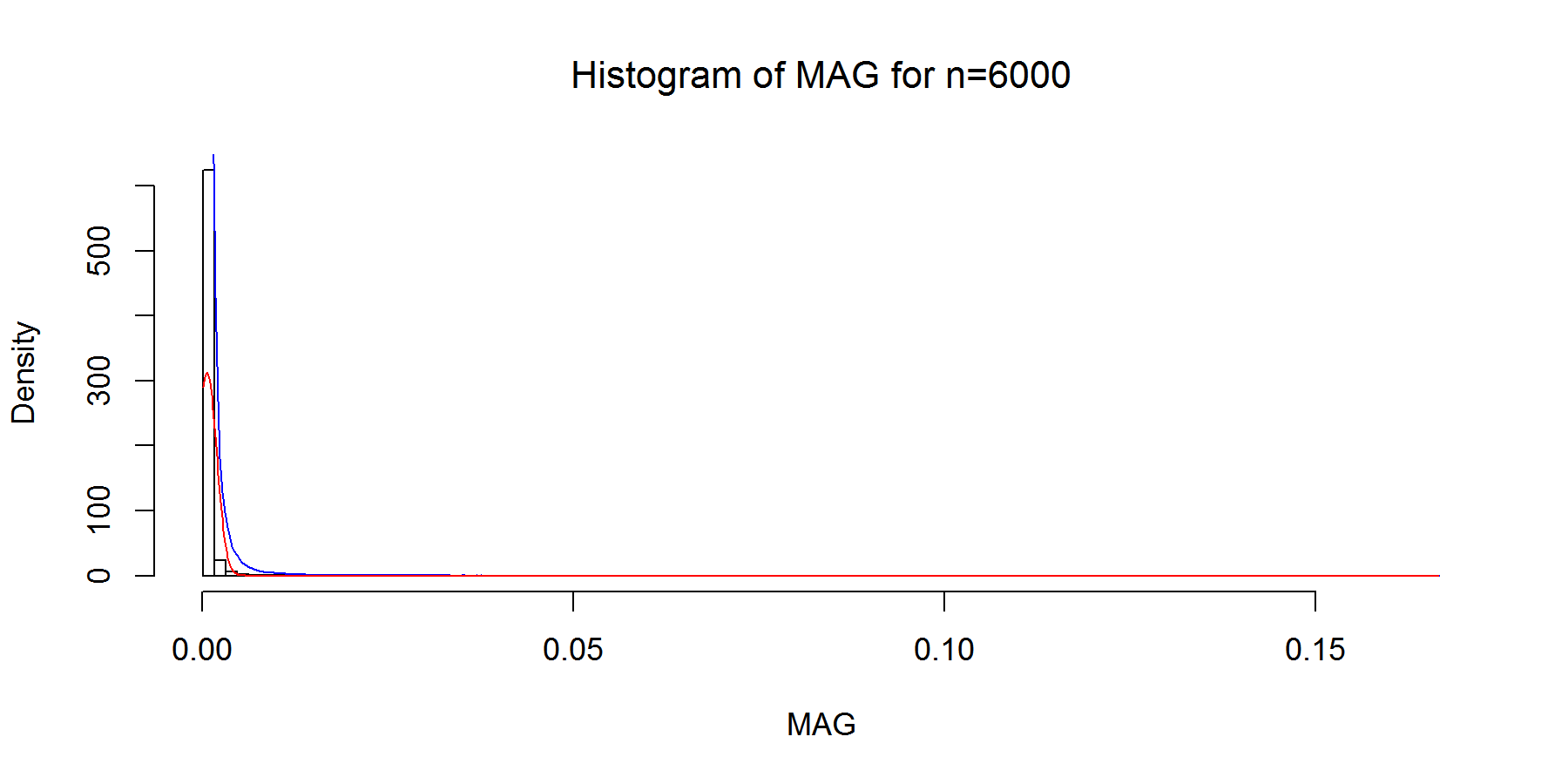


Figure S4 The empirical distribution of $MAG$ under null hypothesis when S=10millions. The blue curve is the fitting kernel density curve and the red curve is the fitting normal distribution curve.


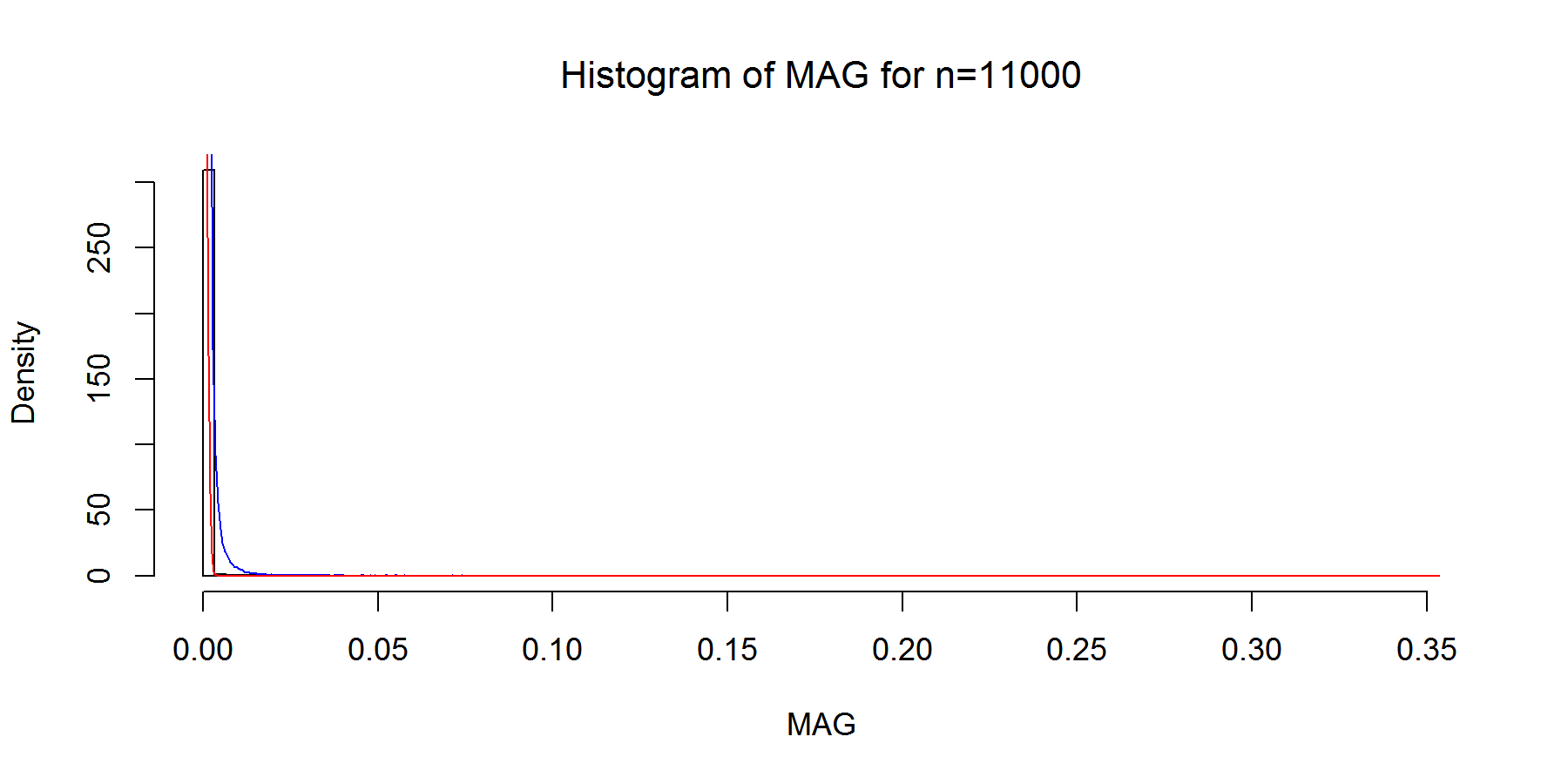


Figure S5 The empirical distribution of $MAG$under null hypothesis when S=10millions. The blue curve is the fitting kernel density curve and the red curve is the fitting normal distribution curve.


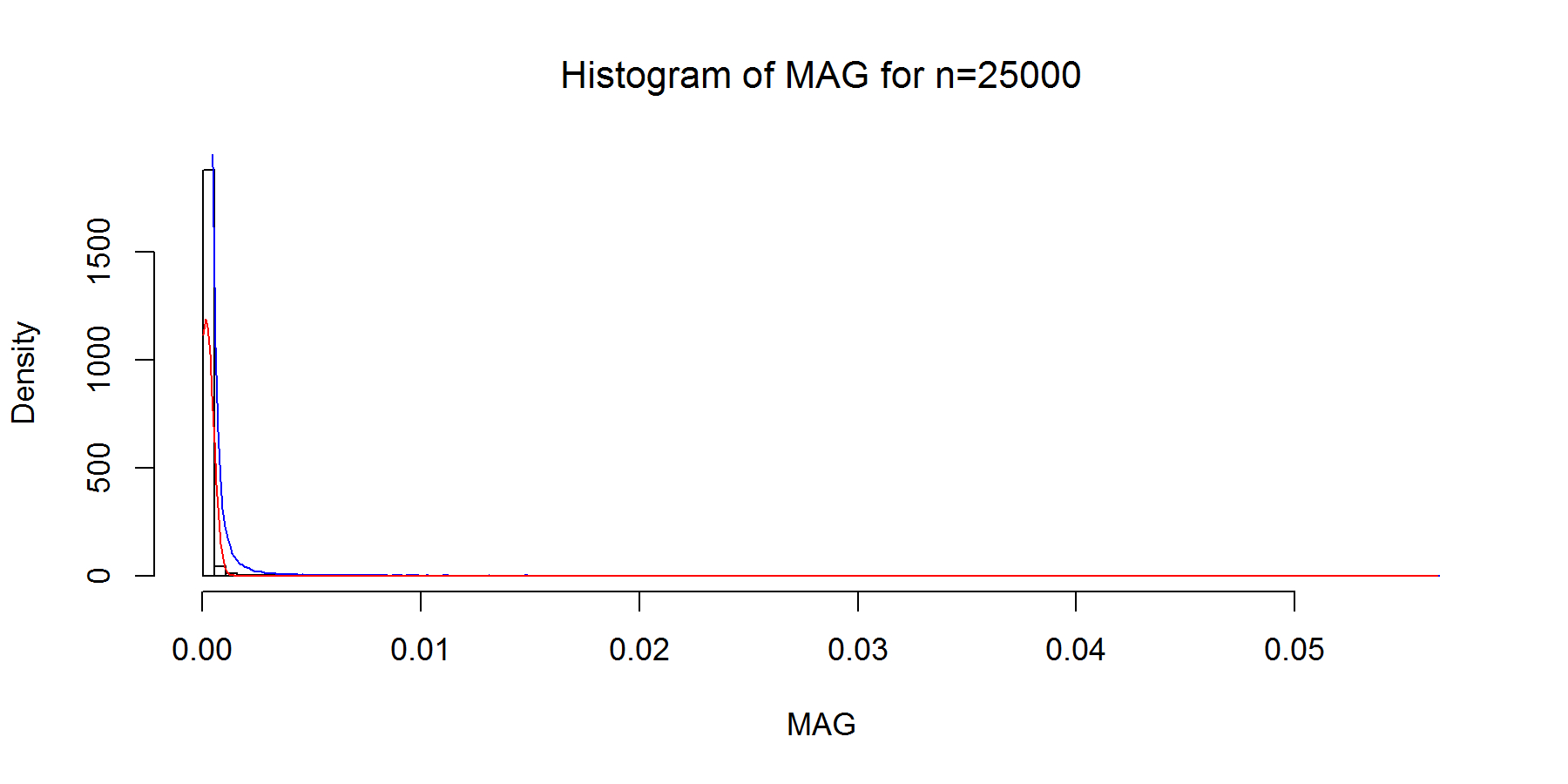


Figure S6 The empirical distribution of $MAG$ under null hypothesis when S=10millions. The blue curve is the fitting kernel density curve and the red curve is the fitting normal distribution curve.





Figure S7 Powers of $EucD$, ${FES}_{0.001}$, ${FES}_{0.01}$,$OrderedList$, $SimSIP$ and ${WeiSumE}^{*}$and when $o = 10$ with 6 scenarios. (A) n=6000, d=10; (B) n=6000, d=20; (C) n=11000, d=10; (D) n=11000, d=20; (E) n=25000, d=10; (I) n= 25000, d=20. The arrangement of variance σ^2^ on *x* axis is a series (0.2, 0.25, 0.3, 0.35) on which the power of six measures can be ranged from 0.1 to 0.95. $FES0.001$ represents measure ${FES}_{0.001}$ and $FES0.01$represents ${FES}_{0.01}$.





Figure S8 Powers of $EucD$, ${FES}_{0.001}$, ${FES}_{0.01}$,$OrderedList$, $SimSIP$ and ${WeiSumE}^{*}$and when $o = 20$ with 3 scenarios. (A) n=6000, d=20; (B) n=11000, d=20; (C) n= 25000, d=20. The arrangement of variance σ^2^ on *x* axis is a series (0.3, 0.35 0.4, 0.45) on which the power of six measures can be ranged from 0.1 to 0.95. $FES0.001$ represents measure ${FES}_{0.001}$ and $FES0.01$represents ${FES}_{0.01}$.


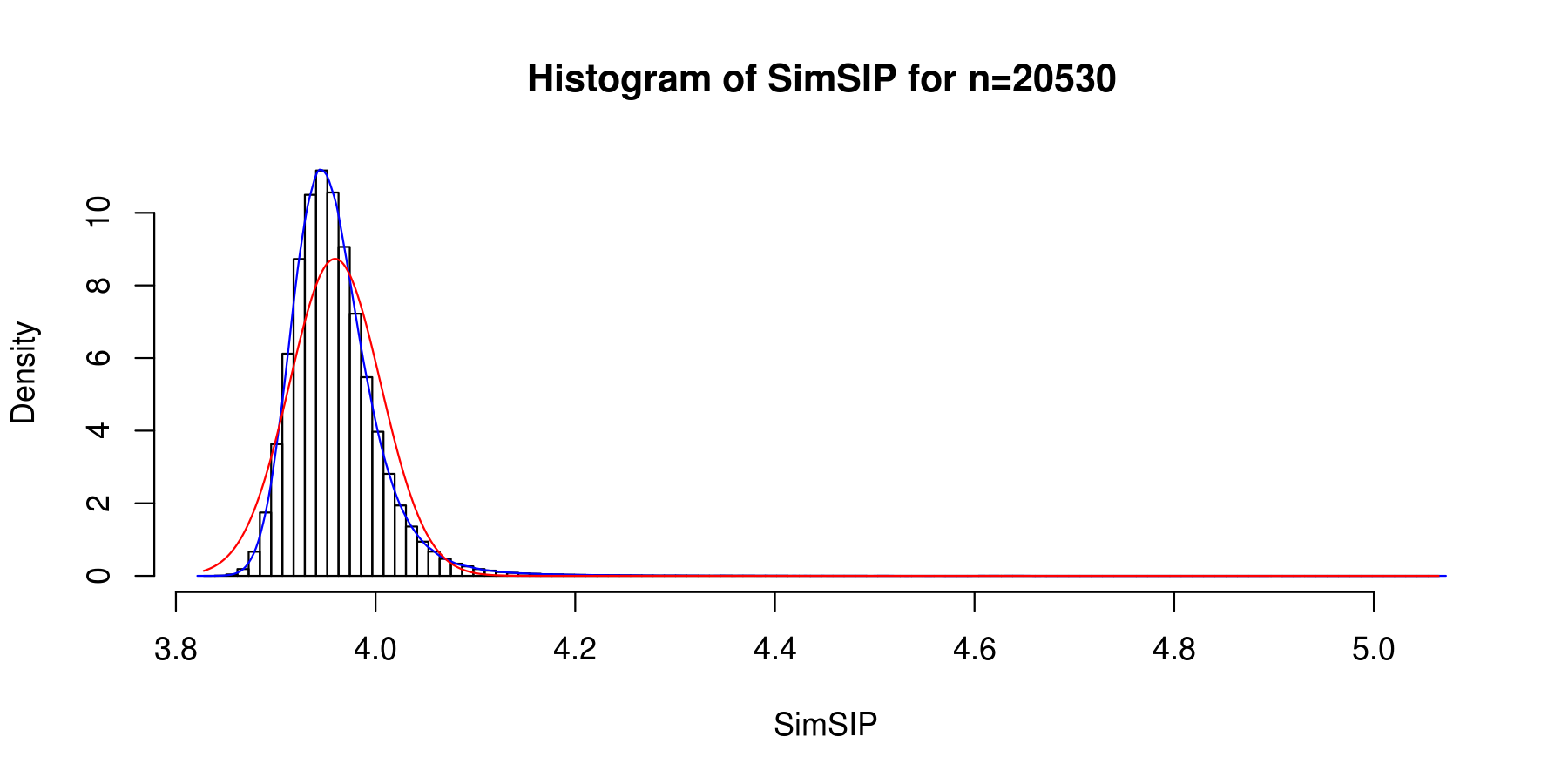


Figure S9 The empirical distribution of $SimSIP$ under null hypothesis when S=10 [million](javascript:;)s. The blue curve is the fitting kernel density curve and the red curve is the fitting normal distribution curve ( Note 20530 is the number of genes in TCGA).


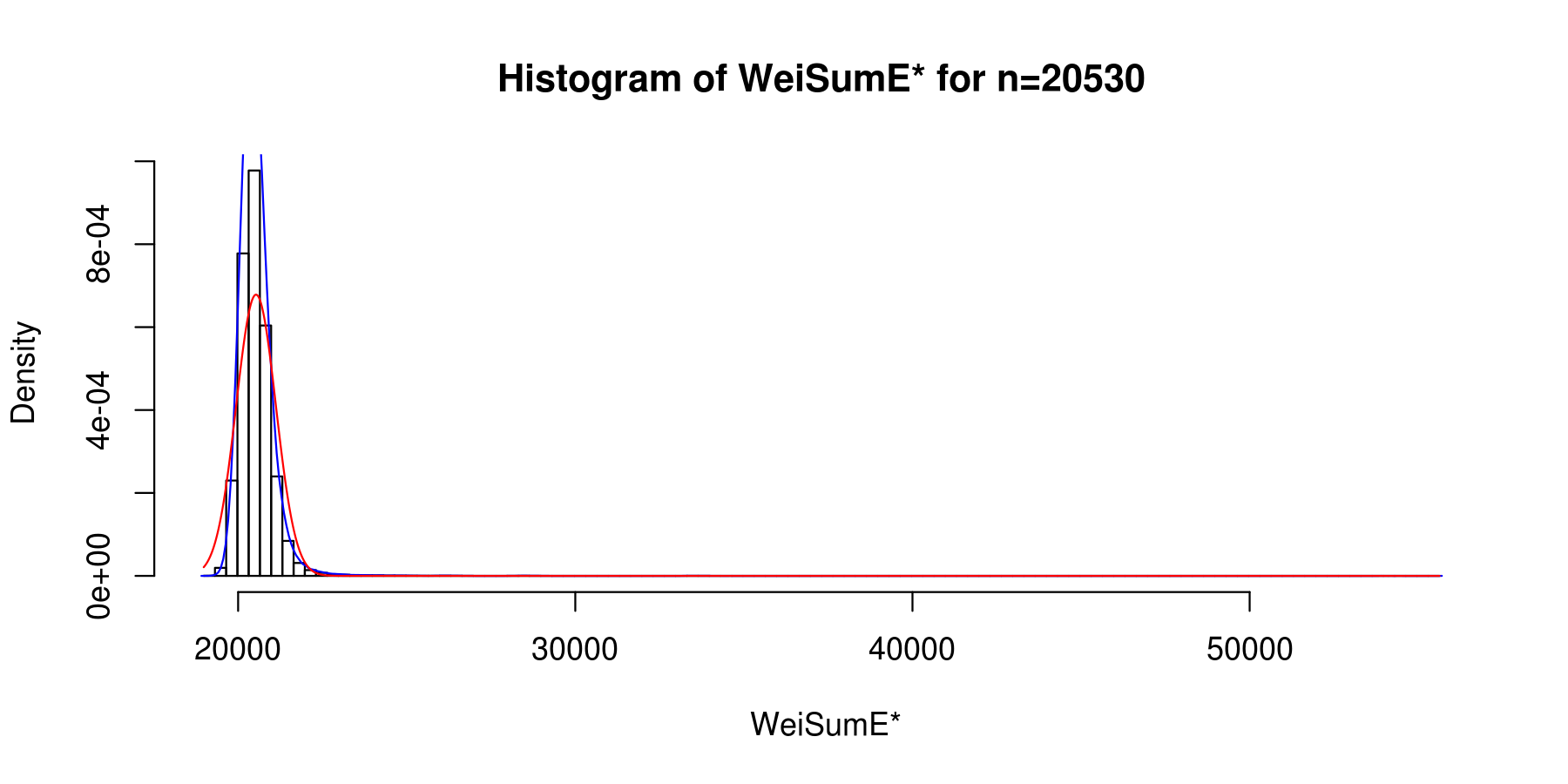


Figure S10 The empirical distribution of ${WeiSumE}^{*}$under null hypothesis when S=10 [million](javascript:;)s. The blue curve is the fitting kernel density curve and the red curve is the fitting normal distribution curve ( Note 20530 is the number of genes in TCGA).


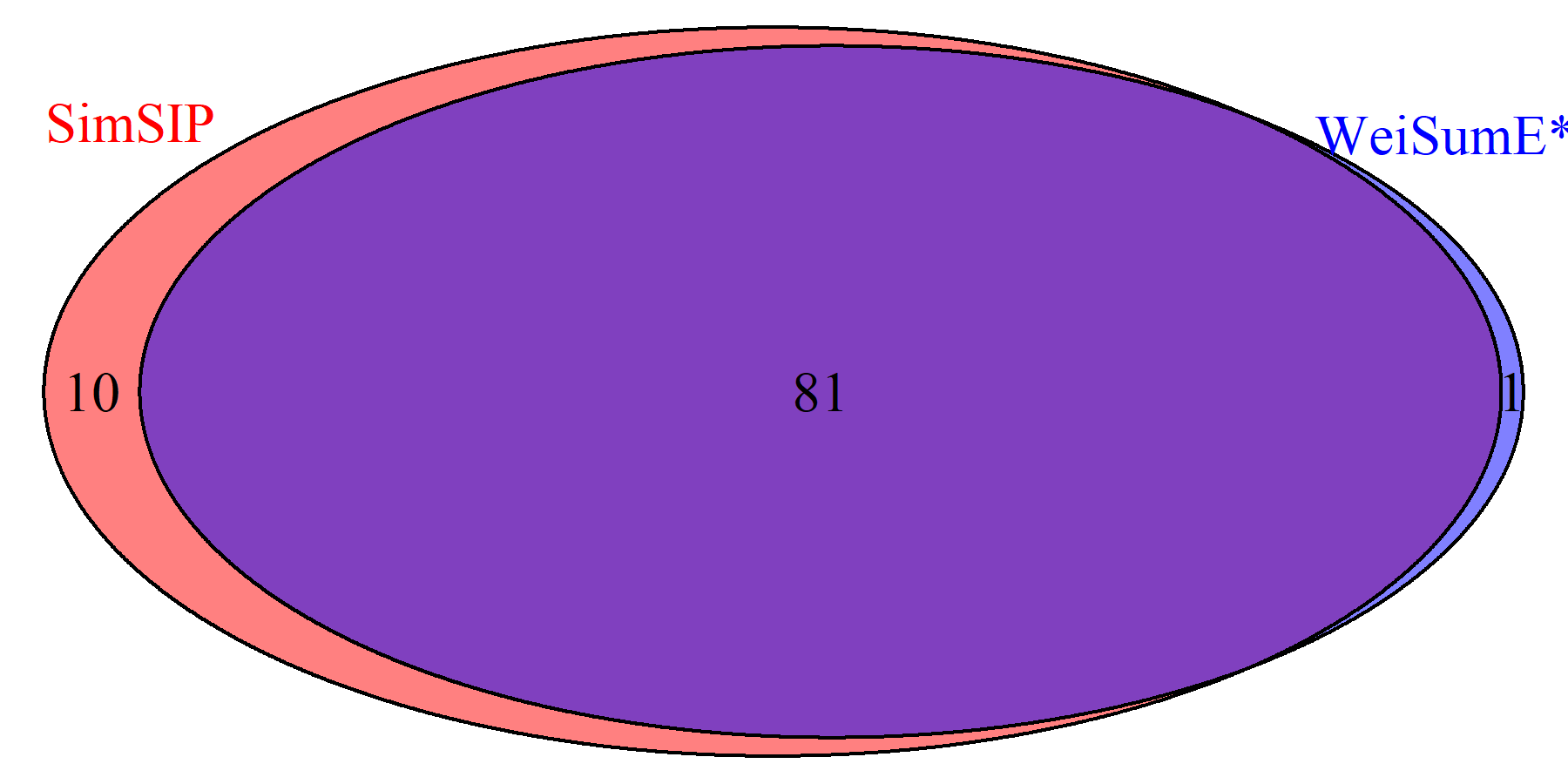


Figure S11 The Venn diagram of significant cancer pairs found with $SimSIP$ and ${WeiSumE}^{*}$*.* There are 91 significant cancer pairs in$SimSIP$set , 82 significant cancer pairs in ${WeiSumE}^{*}$ set and 81 cancer pairs in their intersection after Bonferroni adjustment of empirical *p* value at 5% nominal significance level. More details are shown in Table S3.


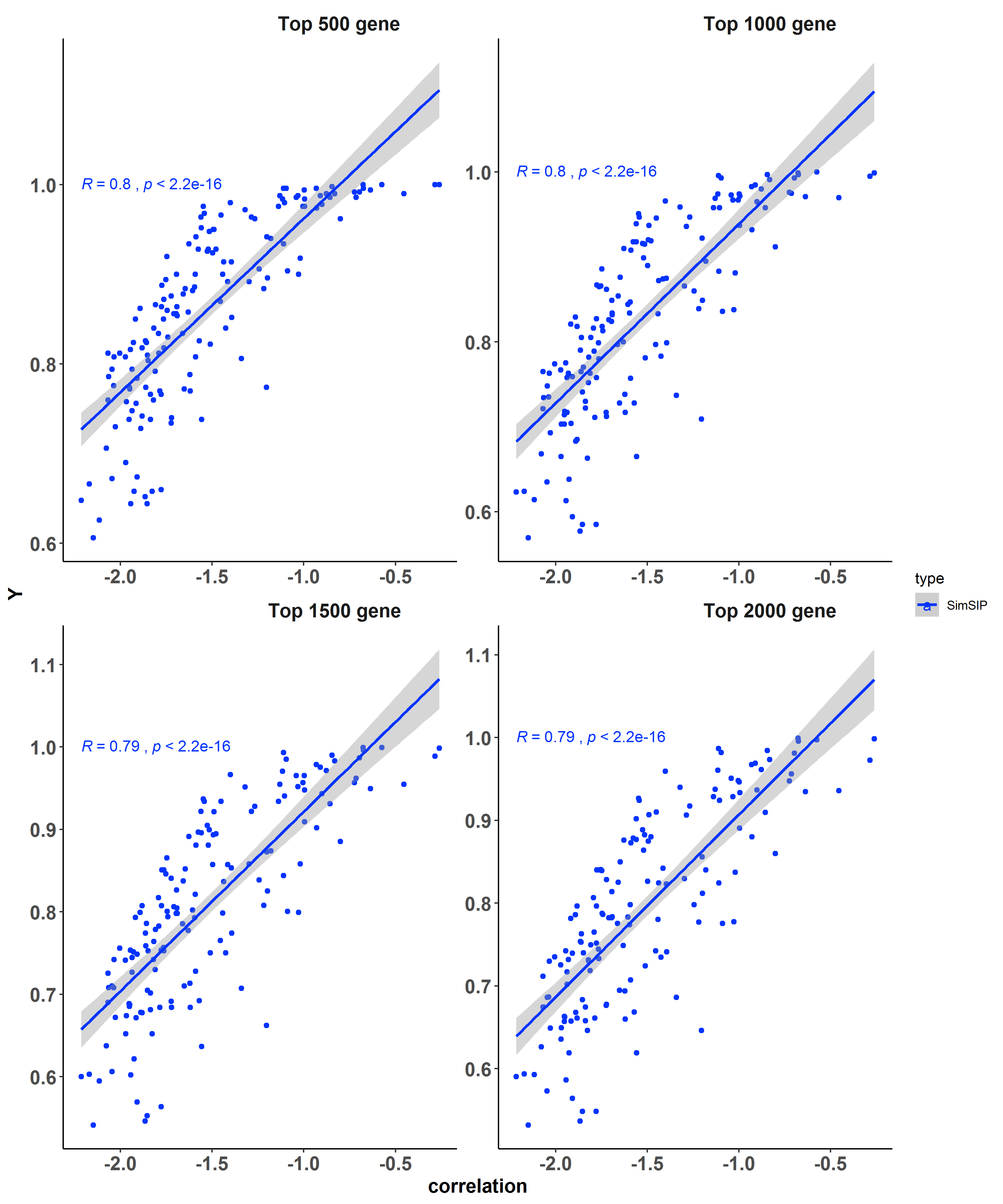


Figure S12 The correlation coefficient between the value of SimSIP and the proportion of genes regulated in the same pattern in the top N (N=500, 1000, 1500, 2000) genes associated with each of cancer pairs. The value on x axis is the logarithmic transformation of SimSIP, the value on y axis is the proportion of genes regulated in the same pattern.


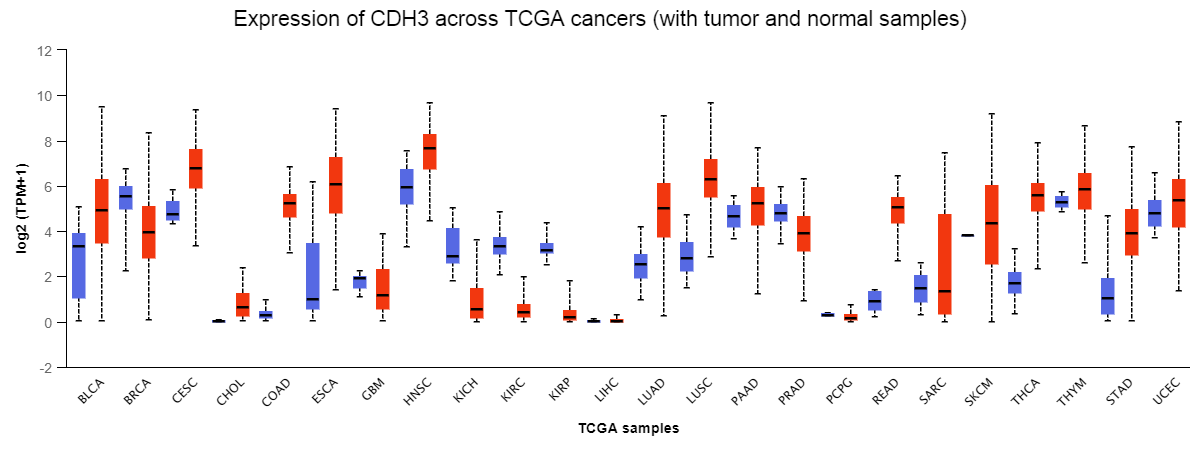


Figure S13Box-Whisker Plot showing gene expression of CDH3in normal and tumor samples acrossTCGA cancers.The blue and red boxplots correspond to gene expression of CDH3 in the control and case groups, respectively. This figure come from UALCAN (<http://ualcan.path.uab.edu/>).


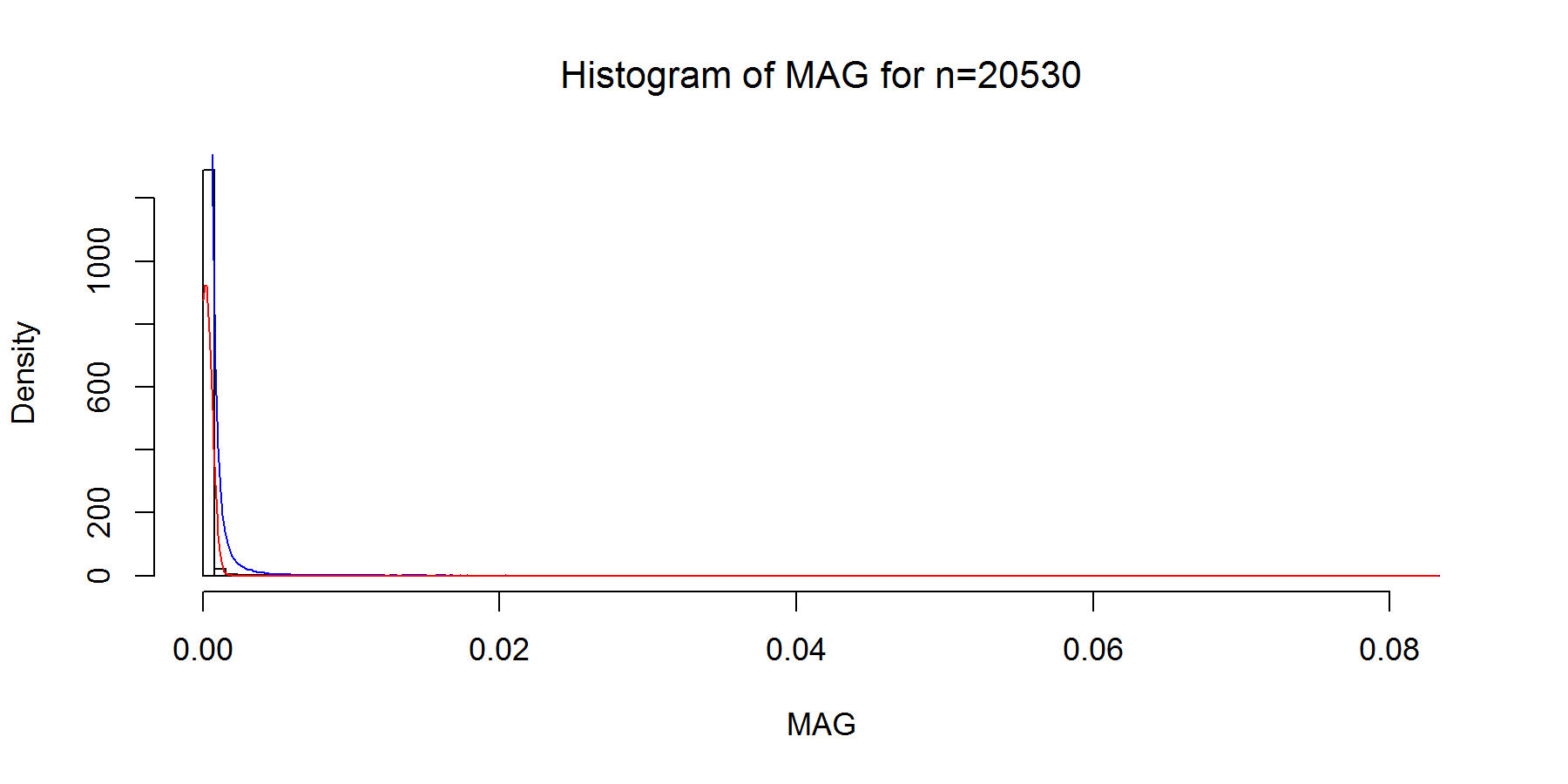


Figure S14 The empirical distribution of $MAG$ under null hypothesis when S=10 millions. The blue curve is the fitting kernel density curve and the red curve is the fitting normal distribution curve ( Note 20530 is the number of genes in TCGA).





Figure S15 Powers of $EucD$, ${FES}_{0.001}$, ${FES}_{0.01}$,$OrderedList$, $SimSIP$ and ${WeiSumE}^{*}$and when $o = 50$, $n=25000$ with 4 scenarios. (A) d=50; (B) d=100; (C) d=200; (D) d=500. The arrangement of variance σ^2^ on x axis is a series (0.1, 0.2, 0.3, 0.4, 0.5, 0.6) on which the power of six measures can be ranged from 0.1 to 0.95. $FES0.001$ represents measure ${FES}_{0.001}$ and $FES0.01$represents ${FES}_{0.01}$.





Figure S16 Powers of $EucD$, ${FES}_{0.001}$, ${FES}_{0.01}$,$OrderedList$, $SimSIP$and ${WeiSumE}^{*}$and when $o = 100$, $n=25000$ with 4 scenarios. (A) d=100; (B) d=200; (C) d=500; (D) d=1000. The arrangement of variance $\sigma^{2}$ on $x$ axis is a series ( 0.2, 0.3, 0.4, 0.5, 0.6, 0.7, 0.8) on which the power of six measures can be ranged from 0.1 to 0.95.$FES0.001$representsmeasure${FES}_{0.001}$and $FES0.01$represents ${FES}_{0.01}$.





Figure S17 Powers of $EucD$, ${FES}_{0.001}$, ${FES}_{0.01}$,$OrderedList$, $SimSIP$and ${WeiSumE}^{*}$and when $o = 200$, $n=25000$ with 4 scenarios. (A) d=200; (B) d=500; (C) d=1000; (D) d=2000. The arrangement of variance $\sigma^{2}$ on $x$ axis is a series (0.01, 0.03, 0.05, 0.07, 0.09, 0.4, 0.5, 0.6, 0.7, 0.8, 0.9) on which the power of six measures can be ranged from 0.1 to 0.95. $FES0.001$ represents measure ${FES}_{0.001}$ and $FES0.01$represents ${FES}_{0.01}$.
