# Supplementary material for: A novel similarity score based on gene ranks to reveal genetic relationships among diseases
Source: PeerJ. 2021 Jan 6;9:e10576. doi: 10.7717/peerj.10576 (PMC7796663; doi:10.7717/peerj.10576)
Supplement: Supplemental Information 2 [file peerj-09-10576-s002.docx]

Table S1 The empirical type I error rates of six measures ($SimSIP$*,* $WeiSumE$**,*$OrderedList$*,*${FES}_{0.001}$*,* ${FES}_{0.01}$and Euclidean distance $EucD$) at 5% nominal significance level under the null model (*o*=0).

|  |  | n=1000 | | | | n=11000 | | | | n=250000 | | | |
| --- | --- | --- | --- | --- | --- | --- | --- | --- | --- | --- | --- | --- | --- |
| $\sigma^{2}$ | method | d=1 | d=5 | d=10 | d=20 | d=1 | d=5 | d=10 | d=20 | d=1 | d=5 | d=10 | d=20 |
| 0.01 | SimSIP | 0.04 | 0.032 | 0.036 | 0.036 | 0.049 | 0.052 | 0.046 | 0.033 | 0.056 | 0.04 | 0.043 | 0.034 |
|  | WeiSumE* | 0.042 | 0.044 | 0.032 | 0.025 | 0.049 | 0.042 | 0.036 | 0.029 | 0.05 | 0.047 | 0.042 | 0.041 |
|  | OrderedList | 0.048 | 0.043 | 0.027 | 0.034 | 0.058 | 0.054 | 0.037 | 0.028 | 0.066 | 0.055 | 0.049 | 0.042 |
|  | FES_0.001_ | 0.055 | 0.052 | 0.05 | 0.059 | 0.053 | 0.034 | 0.066 | 0.051 | 0.054 | 0.04 | 0.047 | 0.047 |
|  | FES_0.01_ | 0.056 | 0.04 | 0.042 | 0.041 | 0.05 | 0.051 | 0.047 | 0.043 | 0.054 | 0.057 | 0.046 | 0.052 |
|  | EucD | 0.04 | 0.055 | 0.049 | 0.041 | 0.035 | 0.044 | 0.053 | 0.053 | 0.053 | 0.062 | 0.053 | 0.04 |
| 0.05 | SimSIP | 0.049 | 0.04 | 0.045 | 0.024 | 0.05 | 0.045 | 0.034 | 0.041 | 0.048 | 0.047 | 0.036 | 0.048 |
|  | WeiSumE* | 0.047 | 0.038 | 0.048 | 0.012 | 0.046 | 0.037 | 0.041 | 0.028 | 0.055 | 0.042 | 0.034 | 0.04 |
|  | OrderedList | 0.041 | 0.042 | 0.043 | 0.032 | 0.048 | 0.037 | 0.044 | 0.032 | 0.059 | 0.063 | 0.054 | 0.05 |
|  | FES_0.001_ | 0.044 | 0.041 | 0.051 | 0.048 | 0.061 | 0.064 | 0.052 | 0.048 | 0.051 | 0.049 | 0.05 | 0.06 |
|  | FES_0.01_ | 0.049 | 0.04 | 0.059 | 0.034 | 0.052 | 0.037 | 0.038 | 0.045 | 0.054 | 0.041 | 0.043 | 0.039 |
|  | EucD | 0.045 | 0.039 | 0.064 | 0.039 | 0.059 | 0.044 | 0.062 | 0.05 | 0.05 | 0.046 | 0.057 | 0.054 |
| 0.1 | SimSIP | 0.048 | 0.053 | 0.052 | 0.029 | 0.051 | 0.045 | 0.048 | 0.052 | 0.053 | 0.066 | 0.051 | 0.044 |
|  | WeiSumE* | 0.054 | 0.044 | 0.05 | 0.023 | 0.044 | 0.045 | 0.048 | 0.044 | 0.046 | 0.058 | 0.05 | 0.043 |
|  | OrderedList | 0.052 | 0.038 | 0.053 | 0.035 | 0.047 | 0.058 | 0.053 | 0.05 | 0.046 | 0.057 | 0.034 | 0.051 |
|  | FES_0.001_ | 0.052 | 0.061 | 0.046 | 0.054 | 0.055 | 0.052 | 0.048 | 0.052 | 0.061 | 0.064 | 0.048 | 0.053 |
|  | FES_0.01_ | 0.058 | 0.044 | 0.053 | 0.041 | 0.048 | 0.051 | 0.045 | 0.048 | 0.047 | 0.054 | 0.056 | 0.045 |
|  | EucD | 0.065 | 0.067 | 0.041 | 0.051 | 0.053 | 0.048 | 0.057 | 0.049 | 0.064 | 0.049 | 0.056 | 0.048 |
| 0.5 | SimSIP | 0.051 | 0.045 | 0.055 | 0.048 | 0.047 | 0.049 | 0.038 | 0.056 | 0.052 | 0.06 | 0.06 | 0.06 |
|  | WeiSumE* | 0.051 | 0.055 | 0.056 | 0.05 | 0.052 | 0.052 | 0.042 | 0.051 | 0.047 | 0.033 | 0.057 | 0.055 |
|  | OrderedList | 0.059 | 0.061 | 0.07 | 0.054 | 0.065 | 0.063 | 0.045 | 0.054 | 0.048 | 0.048 | 0.047 | 0.057 |
|  | FES_0.001_ | 0.041 | 0.048 | 0.055 | 0.053 | 0.051 | 0.061 | 0.046 | 0.038 | 0.044 | 0.044 | 0.052 | 0.048 |
|  | FES_0.01_ | 0.051 | 0.049 | 0.058 | 0.041 | 0.044 | 0.057 | 0.053 | 0.056 | 0.045 | 0.043 | 0.053 | 0.056 |
|  | EucD | 0.052 | 0.06 | 0.054 | 0.053 | 0.053 | 0.058 | 0.05 | 0.042 | 0.033 | 0.053 | 0.05 | 0.047 |

Table S2 The significant cancer pairs found with ${WeiSumE}^{*}$and $SimSIP$

| Method | Significant cancer pairs **^a^** | | | | | | | | | | |
| --- | --- | --- | --- | --- | --- | --- | --- | --- | --- | --- | --- |
| ${WeiSumE}^{*}$ | (KIRC,KIRP) | (COAD,READ) | | (ESCA,LUSC) | (ESCA,STAD) | (BLCA,UCEC) | (LUSC,UCEC) | (BLCA,ESCA) | (BLCA,LUSC) | (LUAD,LUSC) | (ESCA,UCEC) |
|  | (BLCA,BRCA) | (LUSC,STAD) | | (BRCA,UCEC) | (BLCA,STAD) | (ESCA,HNSC) | (BRCA,ESCA) | (BRCA,LUSC) | (HNSC,LUSC) | (BRCA,HNSC) | (STAD,UCEC) |
|  | (COAD,STAD) | (HNSC,STAD) | | (BRCA,LUAD) | (LIHC,UCEC) | (BLCA,HNSC) | (BLCA,LUAD) | (LIHC,LUSC) | (ESCA,LIHC) | (BLCA,READ) | (HNSC,UCEC) |
|  | (BLCA,LIHC) | (CHOL,LIHC) | | (LUAD,UCEC) | (LIHC,STAD) | (READ,STAD) | (BRCA,STAD) | (ESCA,GBM) | (BLCA,COAD) | (GBM,LUSC) | (BRCA,LIHC) |
|  | (LUAD,STAD) | (ESCA,LUAD) | | (GBM,UCEC) | (BLCA,GBM) | (HNSC,LIHC) | (KICH,KIRP) | (LIHC,LUAD) | (COAD,ESCA) | (HNSC,LUAD) | (ESCA,READ) |
|  | (GBM,STAD) | (KICH,KIRC) | | (LUAD,PRAD) | (BRCA,READ) | (CHOL,ESCA) | (GBM,LIHC) | (CHOL,STAD) | (LUAD,READ) | (PRAD,UCEC) | (COAD,LUAD) |
|  | (BRCA,PRAD) | (COAD,LUSC) | | (BLCA,PRAD) | (COAD,HNSC) | (GBM,HNSC) | (CHOL,HNSC) | (BRCA,COAD) | (CHOL,LUSC) | (BRCA,GBM) | (BRCA,CHOL) |
|  | (BLCA,CHOL) | (CHOL,UCEC) | | (HNSC,READ) | (BLCA,THCA) | (READ,THCA) | (READ,UCEC) | (KIRP,LUAD) | (LUSC,READ) | (PRAD,READ) | (COAD,LIHC) |
|  | (KIRP,READ) | (BRCA,KIRP) | |  |  |  |  |  |  |  |  |
| $SimSIP$ | (COAD,READ) | (KIRC,KIRP) | | (ESCA,LUSC) | (ESCA,STAD) | (LUSC,UCEC) | (BLCA,UCEC) | (LUAD,LUSC) | (BLCA,ESCA) | (BLCA,LUSC) | (ESCA,UCEC) |
|  | (LUSC,STAD) | (BLCA,BRCA) | | (BLCA,STAD) | (BRCA,LUSC) | (ESCA,HNSC) | (BRCA,ESCA) | (BRCA,UCEC) | (HNSC,LUSC) | (COAD,STAD) | (STAD,UCEC) |
|  | (BRCA,HNSC) | (HNSC,STAD) | | (BRCA,LUAD) | (LIHC,LUSC) | (LIHC,UCEC) | (BLCA,HNSC) | (BLCA,READ) | (HNSC,UCEC) | (READ,STAD) | (BRCA,STAD) |
|  | (ESCA,LIHC) | (CHOL,LIHC) | | (BLCA,LUAD) | (LUAD,UCEC) | (BLCA,COAD) | (LIHC,STAD) | (ESCA,GBM) | (BLCA,LIHC) | (GBM,LUSC) | (KICH,KIRP) |
|  | (KICH,KIRC) | (ESCA,LUAD) | | (LUAD,STAD) | (BRCA,LIHC) | (COAD,ESCA) | (GBM,UCEC) | (BLCA,GBM) | (HNSC,LIHC) | (ESCA,READ) | (LIHC,LUAD) |
|  | (GBM,STAD) | (HNSC,LUAD) | | (CHOL,ESCA) | (PRAD,UCEC) | (CHOL,LUSC) | (COAD,HNSC) | (BRCA,READ) | (COAD,LUSC) | (CHOL,STAD) | (READ,THCA) |
|  | (BRCA,COAD) | (LUAD,PRAD) | | (LUAD,READ) | (BRCA,PRAD) | (BLCA,PRAD) | (GBM,LIHC) | (HNSC,READ) | (READ,UCEC) | (CHOL,UCEC) | (COAD,LUAD) |
|  | (COAD,UCEC) | (GBM,HNSC) | | (BLCA,THCA) | (CHOL,HNSC) | (BRCA,CHOL) | (BRCA,GBM) | (COAD,THCA) | (LUSC,READ) | (BLCA,CHOL) | (PRAD,READ) |
|  | (HNSC,PRAD) | (COAD,LIHC) | | (LUSC,PRAD) | (KIRP,LUAD) | (BRCA,THCA) | (BRCA,KIRP) | (KIRP,UCEC) | (ESCA,PRAD) | (KIRC,PRAD) | (LIHC,READ) |
|  | (KICH,READ) | |  |  |  |  |  |  |  |  |  |

^a^ The significant cancer pair found with ${WeiSumE}^{*}$alone is marked in blue, the significant cancer pairs found with $SimSIP$ aloneare marked in red, and the significant cancer pairs found with both ${WeiSumE}^{*}$and $SimSIP$ are marked in black.

Table S3The significance of difference of expression level of the top 5 genes in *COAD* and *READ* from UALCAN website (http://ualcan.path.uab.edu/)

| Gene | COAD | | READ | |
| --- | --- | --- | --- | --- |
|  | The expression level | Statistical significance^a^ | The expression level | Statistical significance |
| CDH3 | Up | 1.62437E-12 | Up | 1.62448E-12 |
| AADACL2 | Down | 5.19950E-11 | Down | 9.56510E-04 |
| ETV4 | Up | 1.62448E-12 | Up | <1E-12 |
| OTOP2 | Down | 1.09390E-11 | Down | 2.59760E-03 |
| KRT24 | Down | 1.65125E-05 | Down | 7.63128E-05 |

^a^ The significance of difference of gene expression level between normal and tumor samples estimated by Student’s t-test considering unequal variance

Table S4 Gene annotation and pathway analysis for top 5 genes associated with both *COAD* and *READ* which are detected by $MAG$*.*

| Order | Gene symbol | Description | Biological process (GO) | KEGG pathway |
| --- | --- | --- | --- | --- |
| 1 | CDH3 | cadherin 3 | GO:1902910 positive regulation of melanosome transport; GO:0051796 negative regulation of timing of catagen;GO:1902908 regulation of melanosome transport | (hsa04514)Cell adhesion molecules (CAMs) |
| 2 | AADACL2 | arylacetamide deacetylase like 2 |  |  |
| 3 | ETV4 | ETS variant 4 | GO:0045944 positive regulation of transcription by RNA polymerase II;GO:0045893 positive regulation of transcription, DNA-templated;  GO:1903508 positive regulation of nucleic acid-templated transcription | (hsa05202)Transcriptional misregulation in cancer |
| 4 | OTOP2 | otopetrin 2 | GO:1902600 proton transmembrane transport; GO:0098662 inorganic cation transmembrane transport; GO:0015672 monovalent inorganic cation transport |  |
| 5 | KRT24 | keratin 24 | GO:0070268 cornification; GO:0031424 keratinization; GO:0030216 keratinocyte differentiation |  |

Table S5 The computing time of six measures ($SimSIP$*,* $WeiSumE$**,*$OrderedList$*,*${FES}_{0.001}$*,* ${FES}_{0.01}$and Euclidean distance $EucD$)in calculating the degree of genetic overlaps among the 18 cancer types（153 cancer pairs）.

| Method | $SimSIP$ | $WeiSumE$*** | $OrderedList$ | ${FES}_{0.001}$ | ${FES}_{0.01}$ | $EucD$ |
| --- | --- | --- | --- | --- | --- | --- |
| Time (seconds) | 1.961754 | 6.5455 | 298.3124 | 6.61631 | 7.260617 | 0.190528 |
